# Deriving enzymatic and taxonomic signatures of metagenomes from short read data

**DOI:** 10.1186/1471-2105-11-390

**Published:** 2010-07-22

**Authors:** Uri Weingart, Erez Persi, Uri Gophna, David Horn

**Affiliations:** 1 School of Physics and Astronomy, Tel Aviv University, Tel Aviv 69978, Israel; 2 Department of Molecular Microbiology and Biotechnology, Tel Aviv University, Tel Aviv 69978, Israel

## Abstract

**Background:**

We propose a method for deriving enzymatic signatures from short read metagenomic data of unknown species. The short read data are converted to six pseudo-peptide candidates. We search for occurrences of Specific Peptides (SPs) on the latter. SPs are peptides that are indicative of enzymatic function as defined by the Enzyme Commission (EC) nomenclature. The number of SP hits on an ensemble of short reads is counted and then converted to estimates of numbers of enzymatic genes associated with different EC categories in the studied metagenome. Relative amounts of different EC categories define the enzymatic spectrum, without the need to perform genomic assemblies of short reads.

**Results:**

The method is developed and tested on 22 bacteria for which there exist many EC annotations in Uniprot. Enzymatic signatures are derived for 3 metagenomes, and their functional profiles are explored.

We extend the SP methodology to taxon-specific SPs (TSPs), allowing us to estimate taxonomic features of metagenomic data from short reads. Using recent Swiss-Prot data we obtain TSPs for different phyla of bacteria, and different classes of proteobacteria. These allow us to analyze the major taxonomic content of 4 different metagenomic data-sets.

**Conclusions:**

The SP methodology can be successfully extended to applications on short read genomic and metagenomic data. This leads to direct derivation of enzymatic signatures from raw short reads. Furthermore, by employing TSPs, one obtains valuable taxonomic information.

## Background

Characterizing complex microbial ecosystems remains a challenge for metagenomics. Environments such as soil, containing many thousands of species require massive sequencing power to obtain a reasonable coverage of the microbial community. In practice this means that such studies may suffer from highly incomplete sampling, see for example Tringe et al. [1]. The so called "deep sequencing" technologies offer hope due to their tremendously high-throughput - the Illumina Genome analyzer and the SOLiD 3 (Life Technologies) can currently produce over 10 Gb, and up to 40 Gb of high quality reads, respectively. However these fantastic capacities come with a price - a short read length that currently stands at 100 bases or lower for both these technologies. For a recent review of experimental and computational achievements and challenges in metagenomics see Wooley et al. [2].

Unlike a bacterial genome, where short reads can be compensated for by using paired ends and relying on assembly, a highly complex metagenome will often not enable such assembly, and the short individual reads will therefore constitute the data from which information has to be extracted. Of course, getting significant BLAST hits with queries of 100 nucleotides or below is challenging, which results in no match that can be assigned a putative function for the vast majority of sequence reads. In the seminal paper by Dinsdale and coworkers [3] using reads of 105 bases and below, most of the biomes investigated yielded less than 20% BLAST hits, many of which could not be ascribed a function.

Conventionally, one first tries to reconstruct a long contig from short reads. The contigs are then analyzed for open reading frames (ORFs) which may be translated into putative proteins. The functionality of the putative proteins can be deduced by comparing them with known proteins whose sequence similarity is high enough (e.g. very low BLAST e-values) to warrant such predictions. This can be improved by combining various methods such as studying both phylogeny and function [4]. The problems of handling and analyzing these environmental data have been recently discussed by Raes and Bork [5].

We propose to forego some of the stages used in conventional analysis and consider the multitude of available short reads directly. This can allow us to gather ***inclusive ***information. We use this term to imply functional information on the aggregate of all data rather than the ***exclusive ***information specifying what are the exact genes present and to which species these genes belong. Here we present such a tool employing peptide-based enzymatic signatures and demonstrate its application to quality control and functional investigation of metagenomic data.

Extending the peptide-based approach, we can also derive taxonomic signatures from metagenomic short reads. Current technologies for estimating microbial phylogenetic diversity of metagenomes involve calculation of similarity between sequences encoding rRNAs to database entries such as the ones available in the Ribosomal Database Project, RDP [6]. This procedure requires the expensive operation of assembly of contigs, and is based on the premise that 16S rRNA sequences provide a suitable basis for taxa-separations, defining operational taxonomic units (OTUs) [7]. Our approach differs from this conventional method in two respects: first we deal directly with short reads, second we do not employ the 16S rRNA as the taxonomic indicator. Instead we use SPs of aminoacyl tRNA synthetases (aaRS) for taxonomic indication.

Recently, the algorithm of CARMA [8] was introduced to provide phylogenetic classification directly from short reads. It is composed of two components: detection of Pfam domain and protein family fragments (EGTs) that are conserved in an environmental sample and reconstruction of a phylogenetic tree for each matching Pfam family. The authors state that environmental gene tags as short as 27 amino acids can accurately be classified with high specificity. We provide an accurate alternative to this approach, based on peptides of lengths 7 amino acids and higher, and therefore more suitable for short read data.

The workflow of our paper is the following:

a. Based on the concept of Specific Peptides (SPs) we propose their direct application to short read (SR) analysis.

b. We derive factors that reflect the ratio between counts of SPs, corresponding to a specific EC category, on a set of SRs of a genome or a metagenome, and the numbers of enzyme sequences carrying the same EC annotation on the genome or the metagenome. This is exemplified first on *Escherichia coli *data and further developed on artificial metagenomes of known bacteria, relying on their genomic sequences and enzymatic annotations of their proteins in Uniprot.

c. We develop the concept of TSPs, taxa-specific SPs, using amino-acyl tRNA synthetases that are known to appear only once per species. The determinations of which SPs are taxon-specific, and their associated factors, are derived from all enzymatic data of Swiss-Prot.

The methodology is explained in detail in the following section, and then exemplified and tested in the Results sections.

## Methods

### The Specific Peptides Approach

Kunik et al. [9] have extracted very short (~8aa) deterministic motifs, named Specific Peptides (SPs), whose presence in the protein sequence is a good marker for enzymatic functions. The use of motifs has a long history in bioinformatics [10,11]. It is only recently, however, that the increasing amounts of annotated protein data, combined with novel motif-extraction techniques [12], allowed extracting short SPs and using them with good precision and recall values. SPs are strings of amino-acids, extracted from enzyme sequences using the motif extraction algorithm MEX [12]. They are selected for their specificity to levels of the Enzyme Commission (EC) 4-level functional hierarchy. Weingart et al [13] have demonstrated how SPs can be employed for Data Mining of Enzymes (DME) on any given ensemble of protein sequences. Their methodology relies on coverage length (L, overall number of amino-acids) of SP hits that carry the same EC assignments. In their analysis, L ≥ 7 has led to highly accurate results. They have also updated the SP list, extracting them from a training set of Swiss-Prot data dated July 27th, 2009. This set includes 257,598 SPs of length ≥ 7 with labels corresponding to EC levels 3 and 4. The latter are further filtered for redundancy to discard any SP that contains within it a shorter SP with the same EC specification. This leaves us with a final set of 148,395 SPs that we use in our analysis. Testing the DME approach on a set of 19,849 enzymes that were integrated into Swiss-Prot from July 28th until September 22nd 2009, Weingart et al [13] obtained precision of 99.2% and recall of 92.4%, thus vouching for the high quality of DME predictions at the 3^rd ^level of the EC hierarchy.

Here we propose using an SP search on raw Short Read (SR) data, independent of gene reconstruction. Available reads of *k *nucleotides, where 50 *≤ k *≤ 200, may be turned into peptide candidates in six possible ways, counting 3 possible ORFs and 2 possible strands. Each of these pseudo-peptides is checked for SP hits. The latter are required to reside completely within the pseudo-peptides and have a length of 7 amino-acids or more. Ignoring shorter matches has proved to reduce considerably the number of false positive hits in various trial runs. This reliance on k = 7 and higher k-mers agrees with the DME methodology of Weingart et al [13].

Given a set of short reads we try to obtain a prediction of the number of enzymes in the different EC categories that are expected to be found in the studied metagenome. For that we have to develop a method that relates the number of SP hits observed on a given ensemble of short reads to the expected number of related genes. We define this ratio as the raw-factor, RF(EC) = (number of SP hits)/(number of enzymatic genes) defined for each EC category. To explain this concept we will first illustrate it on a single organism and then proceed to derive it for suitable metagenomes.

### The SPSR methodology: Training on *Escherichia coli*

**Figure 1:**
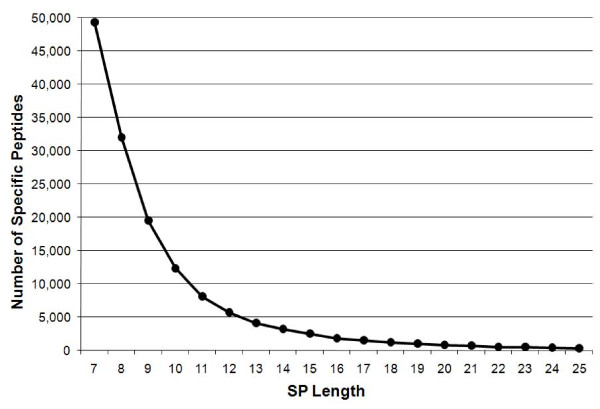
**Distribution of SP lengths**. Distribution of numbers of SPs as function of their length (number of amino-acids).

Here we study the derivation and meaning of factors on *E. coli*, making use of its well-studied genome and its well-annotated genes. We notice that if we insert the full genomic sequence instead of short reads in the evaluation of the RFs, these factors coincide with the average number of SP hits on an enzyme within each EC category. Given the genome, we generate SRs randomly, making sure we obtain a 5-fold coverage of the full genome. Calculating the raw-factors, we realize that they vary as we change the length of our SRs. The RFs for finite short read lengths are always lower than their asymptotic values, because SP lengths have to fit inside the lengths of the SRs. Figure 1 displays the distribution of SP lengths for all EC categories. It allows us to estimate the reduced efficiency of SP detection according to the length of the SR. Thus for a 50 nucleotide short read, no SP hit is expected with length larger than 16 amino-acids. Given this geometrical constraint, the relative efficiency of observation of an SP with length L amino-acids, will be (17-L)/16, just by counting the number of times it can fit into a window of 16 amino-acids. Given the distribution in Figure 1 we estimate the total efficiency for a 50 nucleotide short read to be 0.48. Similarly, we estimate the efficiency for SRs of 100 and 200 nucleotides, to be 0.73 and 0.87 respectively. In practice the numbers may vary somewhat between EC categories, since their SP length distributions are not all equal to one another.

**Figure 2:**
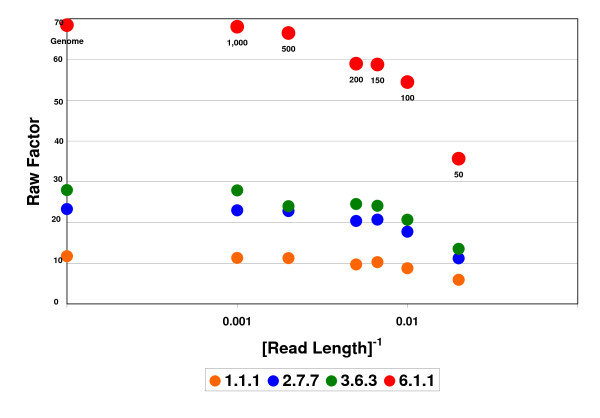
**Raw factors**. Factors defined as (number of SP hits)/(number of enzymatic genes) for *E. coli *on SR ensembles with different lengths (numbers of nucleotides). Displayed are four 3^rd^-level EC categories that possess the largest factors. The x-scale is the inverse of the read-length. The values of the different lengths are denoted within the figure. The extreme points at 1/read-length = 0 correspond to factors evaluated from the full genome length. The latter coincide with values derived from the full proteome.

**Figure 3:**
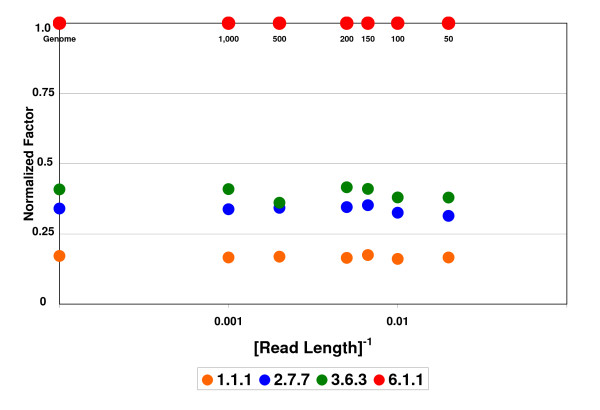
**Normalized factors**. Factors normalized to the 6.1.1. raw-factor for the same data as in Figure 2.

Testing this procedure on *E. coli*, we obtain the factors displayed in Figure 2, following the general trend explained above. The 3^rd ^level EC category with the largest factor is 6.1.1, the aminoacyl tRNA synthetases (aaRS). Since all SPs are subject to similar constraints, we observe that if we measure the relative amounts of different EC categories, as shown in Figure 3, they remain approximately constant as we vary the SR length. We will therefore normalize the raw factors by dividing them by the highest raw factor as follows (Figure 3): NF(EC) = RF(EC)/(RF(6.1.1)). The stability of the NFs will allow us to employ them in metagenomic studies of variable SR lengths.

### The SPSR methodology: Training on 11 bacteria

Next we use a set of 11 bacteria to serve as a training set, to provide factors that are suitable for metagenomic studies. The identities of the bacteria are displayed in Table 1, together with another set of 11 bacteria that will be used as a test set for the resulting factors. The bacteria were chosen from different phyla and classes to provide a balanced representation of the expected variance in metagenomic studies. Moreover, care was taken to choose species with well-studied genomes, having many EC-annotated enzymes. Proteomic information has been derived from Uniprot.

**Table 1:** Bacterial genomes used for training and testing the SPSR methodology

Organism Name	ID	Phyla	Total Uniprot Proteins	Total Uniprot Enzymes	Choice
*Mycobacterium tuberculosis.*	B01	Actinobacteria	5,971	1,371	Train
*Mycobacterium bovis.*	B02	Actinobacteria	3,986	1,253	Test
*Sulfurihydrogenibium azorense*	B03	Aquificae	1,708	486	Train
*Aquifex aeolicus.*	B04	Aquificae	1,556	368	Test
*Cytophaga hutchinsonii*	B05	Bacteroidetes	3,771	895	Test
*Gramella forsetii*	B06	Bacteroidetes	3,554	992	Train
*Pelodictyon luteolum*	B07	Chlorobi	2,078	496	Test
*Chlorobium chlorochromatii*	B08	Chlorobi	1,991	609	Train
*Nostoc punctiforme*	B09	Cyanobacteria	6,601	1,534	Train
*Anabaena variabilis*	B10	Cyanobacteria	5,643	1,362	Test
*Synechocystis sp*	B11	Cyanobacteria	3,529	575	Train
*Bacillus cereus (strain ZK).*	B12	Firmicutes	5,638	1,469	Test
*Bacillus cereus (strain ATCC).*	B13	Firmicutes	5,248	1,546	Train
*Pseudomonas aeruginosa.*	B14	Proteobacteria	9,091	848	Train
*Rhizobium meliloti*	B15	Proteobacteria	7,107	1,583	Test
*Salmonella typhimurium.*	B16	Proteobacteria	5,768	1,279	Train
*Shigella flexneri.*	B17	Proteobacteria	5,395	813	Test
*Salmonella typhi.*	B18	Proteobacteria	5,351	942	Test
*Escherichia coli (K12).*	B19	Proteobacteria	4,412	1,443	Train
*Caulobacter crescentus*	B20	Proteobacteria	3,852	1,238	Test
*Leptospira biflexa*	B21	Spirochaetes	3,730	957	Train
*Thermotoga petrophila*	B22	Thermotogae	1,784	411	Test

Each genome on this list has been randomly divided into reads of length 50, with 5 fold coverage of each genome, and submitted to SP analysis. To gather statistics we have analyzed 15 combinations of 7 out of the 11 organisms of the training set. Each such set of 7 organisms served to define a super-organism (or artificial metagenome) with given annotated enzymes and SP counts. The resulting numbers of SP hits were then compared with the known numbers of enzyme-genes, leading to the desired factors for each EC category. Normalized factors of leading categories are presented in Table 2.

**Table 2:** Factors normalized to the 6.1.1 raw factor, derived from an analysis of SRs with length of l = 50 nucleotides belonging to 15 combinations of 7 out of the 11 organisms of the training set listed in Table 1.

EC	Normalized factor	Standard Deviation
1.1.1	0.15	0.014
1.2.1	0.28	0.024
2.1.1	0.22	0.030
2.3.1	0.11	0.023
2.4.1	0.16	0.028
2.4.2	0.26	0.011
2.5.1	0.25	0.011
2.6.1	0.17	0.010
2.7.13	0.03	0.003
2.7.7	0.45	0.022
3.1.1	0.08	0.022
3.1.3	0.07	0.010
3.2.1	0.07	0.017
3.5.1	0.15	0.034
3.6.1	0.89	0.026
3.6.3	0.45	0.064
4.1.1	0.25	0.019
4.2.1	0.30	0.024
6.1.1	1.00	0.000

### Technical details

We utilize the Knuth Morris Pratt algorithm to perform the search of SPs of length m amino-acids on the six-mode translations of short reads of length n bases. This leads to temporal complexity of order O(m + 2n). Our system runs on a four-processors Intel(R) Xeon(R) CPU 2.33 GHz Linux machine and performs a search of the full SP list on approximately 50,000 nucleotides per hour.

We provide an online web tool that processes short read files provided by users. The system can be accessed at http://horn.tau.ac.il/SPSR.

### Taxon Specific Peptides

The SP methodology can be further developed to characterize taxon-specific SPs, to be denoted as TSPs. This is of interest for pervasive EC categories, some of which we will encounter in our metagenomic analysis. The idea is then, for a particular EC category (6.1.1, aminoacyl tRNA synthetases, aaRS) to filter the SPs according to whether they are specific to a given domain, given phylum or class. The training data on the quoted EC categories are rich enough to allow separation into Archaea, Eukarya and Bacteria, and further specification of bacteria into Proteobacateria, Firmicutes, Cyanobacteria and Actinobacteria. The phylum Proteobacteria, being the largest in the data, allows for further filtering into alpha-, beta- and gammaproteobacteria.

We further concentrate on those aaRS EC numbers that are known to have a single protein per species. An analysis of all bacterial aaRS in Swiss-Prot leads to the statistics displayed in Table 3. Confining ourselves to aaRS that have up to 2% multiple entries, we select the subgroup to be denoted S61 (single proteins in the 6.1.1 EC category), indicated on Table 3. It is this S61 set that we will employ for taxon classification. Eliminating aaRS categories with many multiples helps in reducing the margin of error in our predictions.

**Table 3:** Statistics of bacterial aaRS enzymes in Swiss-Prot data

EC	# doublets	# triplets	# Proteins	% multiples	S61
6.1.1.1	18	0	474	3.80	
6.1.1.2	3	0	125	2.40	
6.1.1.3	1	0	616	0.16	x
6.1.1.4	2	0	703	0.28	x
6.1.1.5	10	0	524	1.91	x
6.1.1.6	60	3	527	12.52	
6.1.1.7	1	0	628	0.16	x
6.1.1.9	0	0	293	0.00	x
6.1.1.10	2	0	421	0.48	x
6.1.1.11	4	0	735	0.54	x
6.1.1.12	2	0	688	0.29	x
6.1.1.13	68	1	172	40.70	
6.1.1.14	276	0	825	33.45	
6.1.1.15	10	0	762	1.31	x
6.1.1.16	14	0	691	2.03	x
6.1.1.17	114	0	808	14.11	
6.1.1.18	0	0	139	0.00	x
6.1.1.19	4	0	675	0.59	x
6.1.1.20	251	0	877	28.62	
6.1.1.21	6	0	627	0.96	x
6.1.1.22	1	0	256	0.39	x

The column '%multiples' refers to the percentage of species that display multiple proteins with the same EC number. The sub-set S61, defined by x entries in the last column, is selected for taxonomic classification.

TSPs are selected for their phylum-level and class-level specificity, after scrutinizing the enzyme data-set of Swiss-Prot. We make use of the same data-set to determine the raw-factors that may be associated with the various TSPs. These would theoretically correspond to very large reads, and only their ratios should be trusted for short reads. Table 4 represents the factors for the S61 subset of the EC category of 6.1.1.

**Table 4:** Raw factors of TSPs corresponding to the S61 subset of EC category 6.1.1, as derived from TSP hits on proteomes in the Enzyme Swiss-Prot data-base

	Taxon	# enzymes	# TSPs	# hits	factor
	Archaea	543	408	1807	3.33
	Eukaryota	259	150	260	1.00
	Bacteria	7752	8310	98556	12.71
Bacteria	Proteobacteria	4341	3768	34376	7.92
Bacteria	Firmicutes	1561	1130	7457	4.78
Bacteria	Cyanobacteria	328	175	541	1.65
Bacteria	Actinobacteria	494	392	1874	3.79
Bacteria	Tenericutes	193	25	72	0.37
Bacteria	Bacteroidetes	132	103	223	1.69
Bacteria	Spirochaetes	185	71	173	0.94
Bacteria	Thermotogae	81	9	22	0.27
Bacteria	Chlamydiae	114	140	383	3.36
Bacteria	Chlorobi	90	31	79	0.88
Archaea	Crenarchaeota	165	53	158	0.96
Archaea	Euryarchaeota	359	281	932	2.60
Proteobacteria	Gammaproteobacteria	2372	1624	13622	5.74
Proteobacteria	Alphaproteobacteria	870	675	3806	4.37
Proteobacteria	Betaproteobacteria	638	394	1950	3.06
Proteobacteria	Epsilonproteobacteria	223	178	430	1.93
Proteobacteria	Deltaproteobacteria	229	9	19	0.08
Firmicutes	Bacillales	614	327	1811	2.95
Firmicutes	Clostridia	374	142	567	1.52
Firmicutes	Lactobacillales	573	387	2543	4.44
Cyanobacteria	Chroococcales	114	64	184	1.61
Bacterioidetes	Bacteroidia	85	67	138	1.62

Shown are all taxa that have more than 80 aaRS enzymes listed in this data-base, numbers of TSP associated with the S61 set corresponding to the relevant taxa, their hits and the deduced raw factors. For bacteria and archaea, predicted numbers of enzymes are assumed to be proportional to numbers of cells present in the sample. In eukaryotes one should apply a further reduction by 1.5, the average number of aaRS enzymes per cell known to be detected in Uniprot data.

We provide an online web tool that processes short read files queried by users, leading to a prediction of relative taxonomic mixtures of the presented data. The system can be accessed at http://horn.tau.ac.il/S61TSPSR.

## RESULTS: Analysis of the Methodology

### Test of the SPSR methodology

**Figure 4:**
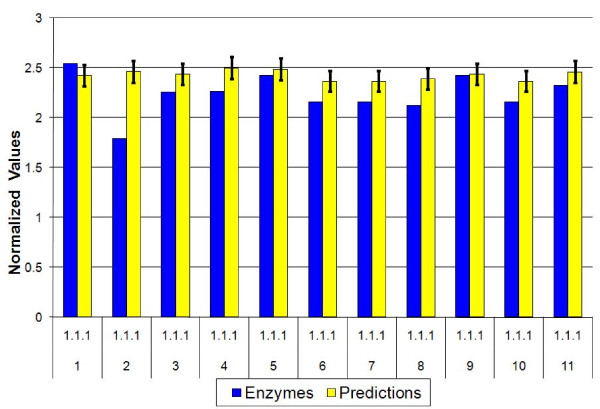
**SPSR test on artificial metagenomes**. SPSR tests, based on normalized factors derived from the training set. Shown are predicted and known numbers of enzymatic genes relative to predicted and known aaRS enzymes (EC = 6.1.1). Error-bars reflect the standard deviations of the factors derived from 15 trials of the artificial super-organisms. Shown here is a comparison of predicted relative amounts of EC = 1.1.1 enzymes for 11 artificial metagenomes containing 7 species from the test set. EC = 1.1.1 is a leading EC category containing alcohol dehydrogenases with NAD^+ ^or NADP^+ ^as acceptor. The results imply a good generalization for metagenomes composed of 7 bacteria.

**Figure 5:**
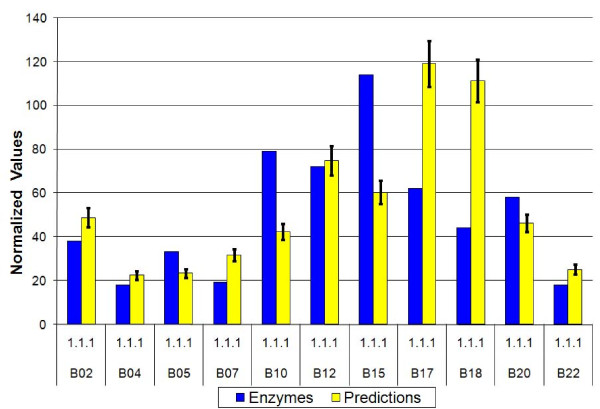
**SPSR test on bacterial genomes**. Predicted relative amounts of EC = 1.1.1 enzymes for single species of the test set show large deviations. Similar results are obtained for single species of the training set.

**Figure 6:**
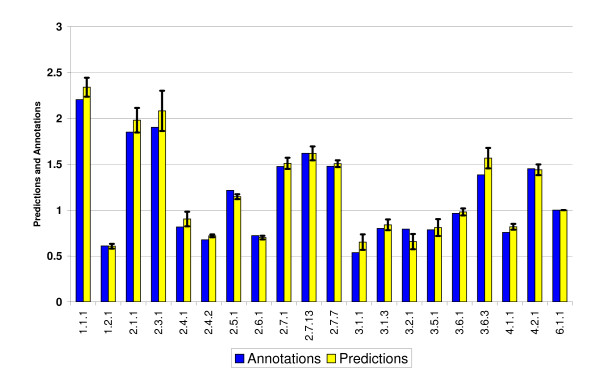
**SPSR test on a metagenome of all 22 bacteria**. Predictions of relative amounts of enzymes belonging to many EC categories, for a metagenome of all 22 bacteria, demonstrate a better fit than the training errors.

In the present section we test the factors derived from the artificial metagenomes (the super-organisms consisting of 7 out of the 11 training set organisms) on the test-set organisms listed in Table 1. Using the errors (standard-deviations) determined by the training procedure, we quote the quality of fits by using the chi-square test, which is expected to be of the order of the number of degrees of freedom, E[(X-μ)^2^/σ^2^] = N (where E is the expectation value, X is the variable whose average is μ and standard-deviation is σ, and N is the number of degrees of freedom). Overall, when the factors are applied to novel artificial 7 species metagenomes, the generalization errors are about the same as expected from the training set errors (see Figure 4), with E ~ 1.5 N. However, when the same factors are applied to single species predictions (see Figure 5) the deviations are much larger. The chi-squared test leads to E ~ 27N. Somewhat better fits are obtained for raw predictions, with E ~ 8N. The poor chi-square values reflect the fact that metagenomic averages smooth-out differences between single organisms. A similar behavior is observed also for single species from the training set. Another aspect of the same effect is seen when larger metagenomes are considered, e.g. one composed of all 22 species, with predictions that are better than the training-set errors shown in Figure 6, where E ~ 0.4 N.

### Test of the TSPSR method at the phylum level

We have applied the S61 TSPs to the 22 bacteria of Table 1. In each case we have calculated the TP (true-positive) signals (i.e. predicted numbers of enzymes associated with the correct phylum) and the FP (false-positive) ones. The results shown in Table 5 validate this methodology. Although some of the data has been included in the training procedure, it should be emphasized that whereas training (i.e. assignment of TSPs) was carried out on all Swiss-Prot enzymes, the calculations of Table 5 are carried out on the full genomes of the 22 organisms, i.e. the procedure includes processing all genic and intergenic regions of these organisms. Precision is defined as TP/(TP + FP). No assignment has been made if the number of predicted enzymes, on the basis of TSPs, was less than 1. This was the case for the two species of Aquificae, for which we have no corresponding TSPs.

**Table 5:** Phylum predictions according to S61 TSPs for the 22 species of Table 1

Organism	Phylum	Precision
*Mycobacterium tuberculosis.*	Actinobacteria	96%
*Mycobacterium bovis.*	Actinobacteria	96%
*Sulfurihydrogenibium azorense*	Aquificae	no prediction
*Aquifex aeolicus.*	Aquificae	no prediction
*Cytophaga hutchinsonii*	Bacteroidetes	74%
*Gramella forsetii*	Bacteroidetes	74%
*Pelodictyon luteolum*	Chlorobi	91%
*Chlorobium chlorochromatii*	Chlorobi	95%
*Nostoc punctiforme*	Cyanobacteria	81%
*Anabaena variabilis*	Cyanobacteria	89%
*Synechocystis sp.*	Cyanobacteria	96%
*Bacillus cereus (strain ZK).*	Firmicutes	94%
*Bacillus cereus (strain ATCC 14579 ).*	Firmicutes	95%
*Pseudomonas aeruginosa.*	Proteobacteria	94%
*Rhizobium meliloti*	Proteobacteria	97%
*Salmonella typhimurium.*	Proteobacteria	99%
*Shigella flexneri.*	Proteobacteria	100%
*Salmonella typhi.*	Proteobacteria	100%
*Escherichia coli (K12).*	Proteobacteria	99%
*Caulobacter crescentus*	Proteobacteria	88%
*Leptospira biflexa serovar Patoc*	Spirochaetes	72%
*Thermotoga petrophila*	Thermotogae	91%

## Results: Environmental Metagenomic Analysis

### Enzymatic signatures of several metagenomes

**Figure 7:**
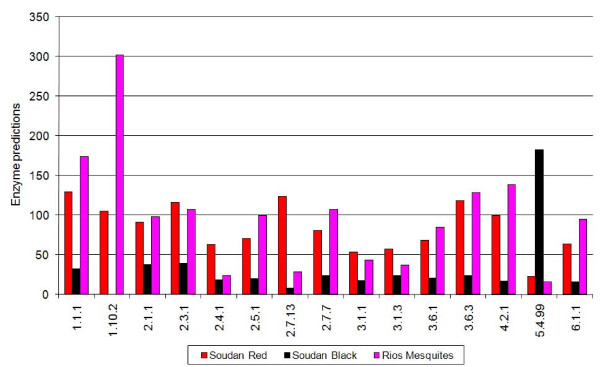
**Enzymatic annotations predicted for three metagenomes**. Predictions of number of enzymes in many EC categories for 3 metagenomes [Dinsdale et al., 2008] derived from SPSR analysis using raw factors for short reads of length l = 100 nucleotides.

Figure 7 displays our analysis of 3 metagenomes taken from Dinsdale et al [3]. All of them comprise short reads, with average lengths of around 100 nucleotides. SPSR predictions are represented in absolute terms, i.e. predicted numbers of enzymes, using the l = 100 raw factors, to exhibit common and different trends among these three examples.

The Rios Mesquites Stromatolites bacteria (to be denoted Rios Mesquitos henceforth) and the Soudan Mine Red biofilm data (to be denoted Soudan Red) have more than 60 predicted proteins in the EC category 6.1.1., while the Soudan Mine Black biofilm (Soudan Black) has only about 20 such proteins. Thus one would conclude that the total coverage content of the first two metagenomes is of the order of three cells or more, while that of the Soudan Black should be only of the order of one cell. In the two large metagenomes we find large contributions of EC category 1.1.1 (alcohol dehydrogenases with NAD^+ ^or NADP^+ ^as acceptor) and EC category 3.6.3 (hydrolases catalysing transmembrane movement of substances). The Rios Mesquitos has the strongest signal in EC 1.10.2, oxidoreductases acting on diphenols with cytochrome as acceptor.

It is clear from Figure 7 that the Soudan Black metagenome is very different from the two others. In particular, it has a very strong signal for 5.4.99., intramolecular transferases. Follow up analysis indicates that this signal is due to 426 SRs that carry the EC 5.4.99.2 (methylmalonyl-CoA mutase) signature. Their identification is due to two SPs with this assignment, NSISISGYH occurring 276 times, and ISISGYHMQEAG occurring 185 times in these data. As these peptides partly overlap, there exist many short reads on which the two occur together. These results stand out for several reasons: their numerous counts outnumber all other enzyme classes by more than an order of magnitude; no other SP of the same EC category is observed in the data; extending these SRs by other partially overlapping short reads does not lead to considerably larger putative proteins. These lines of evidence hint that the Soudan Black data-set should be reexamined, as some artifact has likely been introduced at some point. While such an examination is outside the scope of this paper, we wish to emphasize that the SPSR methodology quickly highlights such anomalies and can therefore serve, among other purposes, also as a rapid quality assessment tool for metagenomic data.

### Taxonomic analysis of metagenomes using TSPs

Taxonomic analysis of the three metagenomes analyzed above has been carried out using S61 TSPs. In all of the metagenomes examined we conclude that Bacteria are the dominant kingdom (with small traces of Archaea in the Soudan mine data). Both Soudan Red and Rios Mesquitos show that, among Bacteria, there is an order of magnitude difference in the quantities of Proteobacteria *vs *Firmicutes. Soudan Black data have the same order of magnitude for both, but given the artifact we have noted, this estimate should be taken with a grain of salt. Predictions for classes of Proteobacteira in Soudan Red are shown in Table 6, where they are compared with the results of the 16S rRNA-based analysis of Edwards et al [14] and with a CARMA analysis [8] of the same data. The Edwards results were estimated from Figure 1 of their paper, and the CARMA analysis was carried out by us using their website. There is an overall agreement regarding relative abundance of alpha- and gamma-proteobacteria, but the details of the minor classes differ among the different methods. This may be because the three methods rely on three different aspects of the data.

**Table 6:** Comparison of class predictions within proteobacteria for the Soudan Red data

Class	Edwards	CARMA	S61TSP
Alphaproteobacteria	40%	37%	45%
Gammaproteobacteria	54%	40%	45%
Betaproteobacteria	2%	8%	8%
Epsilonproteobacteria	0%	2%	2%
Deltaproteobacteria	3%	13%	0%

A fourth metagenomic data-set to which we have applied our taxonomic analysis is that of DeLong et al. [15] who have studied metagenomes in the ocean at different depths, thus obtaining stratified microbial assemblages. The latter have been analyzed according to taxonomic groups, functional gene repertoires and metabolic potential. Their data were assembled into contigs of average length of 1000 nucleotides, and their taxonomic analysis has been carried out by comparing cumulative TBLASTX high-scoring sequence pairs bit scores of each depth against one another. The different depths were grouped into Photic Zone (10 m, 70 m and 130 m) and Deep Water zone (500 m, 770 m and 4000 m). Analysis of these data using S61 TSPs leads to the results displayed in Table 7. Numbers shown are predicted numbers of enzymes in the data. Obviously the quantity of the data amounts to just a few cells in total of all depths. Data are dominated by bacteria although there are some traces of archaea and eukaryotes (with decrease of the latter in deep water). Among bacteria we find a relatively large abundance of Cyanobacteria at low depths (mostly 10 m and 70 m). Proteobacteria, whose fraction in the community is relatively stable as function of depth, may be further analyzed for their breakdown into classes. We find the ratio of Alphaproteobacteria: Gammaproteobacteria: Betaproteobacteria to be 4:3:1 in the photic zone, and 3:4:1 in deep water, i.e. roughly stable with depth (not shown in Table 7).

**Table 7:** Taxonomic predictions of DeLong data based on S61 TSPs

Kingdom	Photic Zone	Deep Water
Archaea	3	3
Eukaryota	1	0
Bacteria	22	32


**Phylum**	**Photic Zone**	**Deep Water**

Proteobacteria	9	12
Firmicutes	1	2
Cyanobacteria	8	2
Actinobacteria	2	2

Numbers signify expected numbers of S61 aaRS enzymes. The latter are proportional to the numbers of cells of the different taxa in the data.

DeLong et al. [15] have constructed large contigs (average length 1000 nucleotides) that can provide much more specific taxonomic information than our EC 6.1.1 based analysis. Nonetheless the latter is consistent with theirs. The advantage of the TSP analysis is that it allows one to obtain a rough taxonomic breakdown of the microbial community when short reads are the sole source of information.

It should be noted that the raw factors of the TSPs were determined by Swiss-Prot data. Since the latter may be richer in SP hits than yet unassigned proteins that are identified by our methodology, the absolute values quoted in Table 7, being based on these raw factors, should be regarded as lower bound estimates of the true taxonomic distribution.

## Conclusions

The use of Specific Peptides allows deriving enzymatic information directly from short reads of genomic and metagenomic data. This is of great importance in view of the large amount of data-analysis performed with short read methods. It is of particular importance in metagenomic studies, where the organismal composition of the studied data is usually unknown and contig assembly is often impossible. Thus one may functionally study high complexity ecosystems, such as soil and seawater, overcoming the barrier of genome reconstruction, by deriving enzymatic signatures in a straightforward manner.

The enzymatic signatures obtained may serve for coarse grain functional characterization of the environment. Lapierre and Gogarten [16] have pointed out that "character genes" typical to taxonomic groups, such as methanogen-specific enzymes, may also inform us of the composition of the microbiome. We have shown that the use of TSPs for aaRSs, can serve as the basis for taxonomic analysis. Our SP signatures can also serve as indicators for novel functionalities and, in extreme cases, as indicators for the possible contamination of the data-set that is being analyzed.

We provide a webtool at http://horn.tau.ac.il/SPSR that analyzes sets of short reads, extracting all those that have SP hits, together with the indication of their EC categorization. These lists can be further processed, by the tools explained above, to provide enzymatic spectra, or to search for consistency of the analyzed data.

The aaRS super-family plays a special role in our analysis because of several reasons. The first is the large over-all similarity of aaRS enzymes throughout all kingdoms of life, leading to extraordinarily large numbers of 6.1.1 SPs derived from the training-set. This is reflected by the large factors of the 6.1.1 category in Figures 2 and 3. The second reason is their usefulness in discriminating among species, by providing a large number of TSPs. Finally, the fact that for many of the aaRS enzyme types there exists one corresponding protein on each bacterial genome, allows using this super-family as a suitable calibrating device.

Our use of the aaRS SPs as taxonomic measures can be compared to the phylogenetic classification based on Environmental Gene Tags (EGTs) introduced by Krause et al [8] in their CARMA tool. Their method is based on selecting DNA fragments of lengths of order 100 bases, i.e. short reads, and comparing them to Pfam profile HMMs. The identified short reads are defined as EGTs. Incorporating them into phylogenetic trees, the authors developed an algorithm that provides a taxonomic distribution with relatively high accuracy. The similarity between the two tools is that both depend on protein-markers rather than on 16S rRNA ones, which is the gold standard of prokaryotic taxonomy.

There are however many differences. First, they employ Pfam domains over many protein families, whereas we concentrate on SPs of aaRS enzymes only. This guarantees that their tool is more powerful, in the sense that its larger statistics allows for extension to lower taxonomic levels than ours. Second, their methodology relies on employing a battery of tools of the trade, such as BLASTX for sequence matching, pHMM for the Pfam generated ETGs, and PHYLIP for clustering phylogenetic trees. This is commonly regarded necessary, in order to take into account all the generated know-how in bioinformatics. We, on the other hand, rely on a simple look-up table of SPs that has been generated from the enzymes that exist on Swiss-Prot. Its advantage is its simplicity. Third, both methods suffer from biases, since their tools are constructed on existing labeled data. CARMA provides its final results by counting the number of EGTs correlated with each taxon. The analog in our case would have been to count the TSPs. Because of the simplicity of our approach we are aware of one explicit bias: TSP hits differ among taxa because of differences in the sizes of TSP pools. We are able to address this bias by correcting the numbers of TSP counts through the use of raw-factors, providing expected numbers of proteins that should be proportional to numbers of cells. Thus, without diminishing the value of tools like CARMA, we believe that our tool has some clear advantages, and should be used as an additional source of information.

We provide a taxon-search webtool at http://horn.tau.ac.il/S61TSPSR. Upon submission of a list of short reads, it extracts taxonomic distributions at levels of kingdoms, bacterial phyla, and bacterial classes.

### Webtools

Webtool providing SP hits on queried lists of genomic short reads is available at http://horn.tau.ac.il/SPSR.

Webtool providing taxonomic analysis of short read data is available at http://horn.tau.ac.il/S61TSPSR.

## Abbreviations

EC: enzymes commission definition of function; SP: specific peptide; SR: short read; SPSR: the methodology of SP searches on SRs developed here; TSP: taxon specific peptide; S61: subgroup of EC: 6.1.1. enzymes defined in Table 3; RF: raw factor (see definition in Methods); NF: normalized factor (see definition in Methods); MEX: motif extraction method of Solan et al. 2005; DME: data mining of enzymes method of Weingart et al. 2009; aaRS: aminoacyl tRNA synthetases.

## Authors' contributions

UW carried out the extensive data searches, developed the methodology, and applied it to different metagenomes. EP analyzed the marine metagenomic data and participated in development and application of the taxonomic analysis. UG and DH conceived of the study and participated in its design. DH participated in development of the methodology and analysis of the data. UG and DH drafted the manuscript, and all authors read and approved its final form.
